# Impact of inotuzumab ozogamicin as bridging therapy and tumor burden in CAR-T therapy for B-acute lymphoblastic leukemia

**DOI:** 10.3389/fimmu.2025.1725878

**Published:** 2025-12-18

**Authors:** Patricia Alcalde-Mellado, Victoria Ruiz-Maldonado, Javier Delgado-Serrano, Luzalba Sanoja-Flores, Marta Reinoso-Segura, Laura Pérez-Ortega, Belén Sierro-Martínez, Estefanía García-Guerrero, Concepción Pérez de Soto, José María Pérez-Hurtado, José González-Campos, José Antonio Pérez-Simón, Águeda Molinos-Quintana, Teresa Caballero-Velázquez

**Affiliations:** 1 Department of Hematology, University Hospital Virgen del Rocío, Instituto de Biomedicina de Sevilla (IBIS)/Consejo Superior de Investigaciones Científicas (CSIC), Universidad de Sevilla, Sevilla, Spain; 2 Pediatric Unit, Department of Hematology, University Hospital Virgen del Rocío, Instituto de Biomedicina de Sevilla (IBIS)/Consejo Superior de Investigaciones Científicas (CSIC), Universidad de Sevilla, Sevilla, Spain

**Keywords:** acute lymphoblastic leukemia, bridging therapy, CAR-T, flow cytometry, inotuzumab ozogamicin, stem memory T cells

## Abstract

**Introduction:**

Inotuzumab ozogamicin is increasingly being used as bridging therapy (BT) prior to chimeric antigen receptor T-cell (CAR-T) in relapsed/refractory B-cell acute lymphoblastic leukemia (R/R ALL), but its impact on CAR-T expansion and clinical outcomes remains controversial.

**Methods:**

This study analyzed 24 R/R ALL patients receiving tisagenlecleucel after BT (Inotuzumab [n=10] vs. chemotherapy/steroids [n=14]). CAR-T expansion was monitored via multiparametric flow cytometry at different time-points after infusion, with outcomes assessed by event-free survival (EFS), overall survival, and immunophenotypic profiling.

**Results:**

Results indicate that despite lower CAR-T expansion with inotuzumab (median 58.3 vs. 337.7 CAR-T/μL; p=0.011), no difference at 12-month EFS was observed (66.7% vs. 64.3%; p=0.648). However, low tumor burden (LTB) at the time of infusion correlated with improved EFS (81.8% vs. 25% high tumor burden (HTB); p=0.019). Remarkably, inotuzumab achieved superior tumor reduction: 80% of HTB patients achieved LTB vs. 28.6% with chemotherapy (p=0.032). Achieving or maintaining LTB significantly improved EFS in 12 months (81.8% and 100% respectively) vs 25% for patients who maintained HTB. Immunophenotyping revealed a higher proportion of CD8+ stem cell memory (SCM) CAR-T cells at peak of expansion in the inotuzumab group (p=0.036). Interestingly, shorter EFS was observed among those patients who presented a lower percentage of SCM CD8+ CAR-T and lower SCM CD8+CARneg/μL on 28 days and on 90 days after CAR-T therapy.

**Discussion:**

Overall, inotuzumab as BT effectively reduces tumor burden but attenuates CAR-T expansion without compromising survival outcomes. As high tumor burden is a dominant driver of relapse and toxicity, the net effect of inotuzumab may be favorable in selected patients.

## Highlights

- Inotuzumab as bridging therapy effectively reduces tumor burden but attenuates CAR-T expansion without compromising survival outcomes.
- A higher proportion of circulating stem cell memory CD8+ T-cells was observed at the peak of expansion in the inotuzumab group and these cells have been correlated with better prognosis.

## Introduction

In recent years, new treatments have emerged for acute lymphoblastic leukemia B (B-ALL) in patients with relapsed or refractory disease (1–4). Chimeric antigen receptor T-cell (CAR-T) therapy has emerged as a breakthrough therapeutic option for pediatric and young adult patients (up to 25 years of age) with relapse or refractory B-ALL (R/R ALL) (5–7).

Disease burden at the time of CAR-T cell infusion has been recognized as a prognostic factor for patients with ALL (8–10) and, for this reason, different options have been evaluated as bridging therapy (BT) to effectively reduce tumor load until lymphodepletion (LD). Currently, there is no standardized BT, but the choice is individualized according to the hospital and the patient’s clinical situation. In addition to decrease tumor load, it would also be desirable to reduce complications attributable to BT. In this context, high intensity chemotherapy increases the risk of infectious complications without a clear benefit in outcome (11).

BT options include conventional polychemotherapy and immunotherapy (12). The latter group comprise drugs such as blinatumomab, a bispecific anti-CD19/anti-CD3 antibody (13) and inotuzumab ozogamicin, a monoclonal antibody conjugate directed against CD22 that carries a cytotoxic agent that allows internalization into the cell and induces apoptosis of cells expressing this marker (14).

Despite its benefits, the use of immunotherapy as a bridging treatment is controversial. In this sense, Krueger et al. have suggested that the elimination of B-cells induced by these drugs may reduce the antigenic exposure required for optimal activation and expansion of modified CAR-T cells, which could compromise the efficacy of the treatment (15). Accordingly, B-cell depletion prior to CAR-T cell infusion might decrease the persistence and expansion of these CAR-T cells, affecting their ability to eradicate residual disease and prolong remission (16). However, other authors have shown that prior use of immunotherapy does not significantly compromise CAR-T cell expansion or long-term clinical outcomes. In fact, the expansion of CAR-T cells has not been well characterized in this context.

Several studies highlight the relevance of monitoring not only the expansion of CAR-T cells but also the composition of subpopulations to predict therapeutic efficacy. For instance, proportion of CD8+ effector T cells and degree of activation (17), subpopulation of T cells as naive and central memory T cells (18–20) or the identification of CAR-Tγδ^+^ (21) have been related to toxicity and overall outcomes.

With this background, we conducted a study among patients with R/R ALL who have been treated with tisagenlecelucel to explore the prognostic impact of inotuzumab ozogamicin as BT and to characterize CAR-T cell expansion after this treatment.

## Methods

### Study design and patient characteristics

Twenty-four children and young adults with R/R B-ALL who received a single intravenous infusion of tisagenlecleucel in our institution were included consecutively and prospectively for the study of monitoring and characterization of CAR-T expansion between March 2021 and July 2024. All patients met the inclusion criteria of the Spanish state-funded access program, which includes R/R B-ALL after two or more lines of systemic therapy or after transplant. Retrospectively, we proposed to evaluate the influence of bridge therapy in combination with the characterized expansion in these patients. Research was conducted according to the Declaration of Helsinki and was approved by the local institutional ethics committee (CEI VM-VR_03/2021_N; code S2100234). CEI de los Hospitales Universitarios Virgen Macarena y Virgen del Rocio). Informed consent was obtained from all subjects. Tisagenlecleucel was infused after LD based on fludarabine (30mg/m2/day for 4 days) and cyclophosphamide (500 mg/m2/day for two days). The data cut-off date was February 2025.

### Definition of response, disease burden, BT and B-cell aplasia *assessment*

Morphological complete remission was defined as ≤5% blasts in bone marrow (BM) with complete recovery (CR) or with incomplete hematologic recovery (CRi) defined by CR but 1 or more of the following: neutrophils less than 1.0 x10^9^/L, platelets less than 100 x10^9^/L, or platelet/blood transfusions within 7 days. Measurable residual disease (MRD) was evaluated by MFC performed in the local laboratory. MRD were determined by next generation flow (NGF) according to the Euroflow B-ALL MRD protocol on BM samples (22, 23). Relapses were defined as any percentage of BM blasts > 0.01% after CR/CRi beyond day 28 (D28) post-infusion or evidence of extramedullary disease. Disease burden was evaluated prior to LD. High tumor burden (HTB) was defined as >5% of BM blasts by MFC, whereas a count < 5% BM blasts indicated a low tumor burden (LTB). The definition of loss of B-cell aplasia (BCA) included the reappearance of B lymphocytes in peripheral blood (> 1 cell/μl) after absolute -BCA confirmed by an increase in B-cell count over time. BT was administered at physician´s discretion. Inotuzumab subgroup of patients received this treatment as BT between leukapheresis and CAR-T cells infusion and 5 half-lives of washout period (60 days) before CAR-T infusion. ALL Hematotox score was calculated according to the criteria published by Nair et al. (24) using neutrophil, platelet, hemoglobin, C-reactive protein, and tumor burden values ​​before LD.

### Flow cytometry assays

Peripheral blood samples were obtained from the patients on days 5, 7, 11, 14, 21, 28, month +3, month +6, and month +12 after CAR-T cell infusion (**Supplementary Figure 1**). In addition, leukapheresis samples were also collected, which were later used to manufacture the CAR-T cells. For monitoring and immunophenotypic characterization of CAR-T cells a bulk lysis was carried out according Euroflow protocols and stain-lysis-wash protocol as we have previously validated and reported (20). The samples were stained with the antibody and fluorochrome combination listed in the **Supplementary Table 1**. Samples were acquired using BD FACSCanto II™ (FACSDiva software, BD bioscience) and BD FACSLyric™ (FACSSuite software, BD bioscience) cytometers. Data analysis was performed using Infinicyt™ software 2.0.6 (BD bioscience). The analysis strategy used for the characterization of CAR-T and immune subpopulations is described in the **supplementary material**. Stem cell memory (SCM) was identified based on the following phenotype: CD3+, CAR+, CD8+, CD4-, CD45RA+, CD62L+, CD27++, CD95 +. Representative plots of the gating strategy are included in the Supplementary Material (**Supplementary Figures 2–4**). The phenotype used to define each of the populations is detailed in **Supplementary Tables 2, 3 and 4**.

### Statistical analysis

Statistical analyses were performed using SPSS 26 and GraphPad Prism v8. The data did not meet the assumptions required for parametric tests, so the nonparametric Mann-Whitney test was used to calculate the differences in distributions between two groups and the Kruskal-Wallis test to search for differences between three or more groups. CAR-T cell expansion was quantified using the Area Under the Curve (AUC). This metric was calculated from CAR-T/µL data collected by MFC on Days 5, 7, 11, and 14 after CAR-T infusion, according to the formula detailed in the Supplementary Material (**Supplementary Figure 2**). Data from a minimum of three timepoints were required for AUC determination. Cutoff points for variables were calculated using medians or ROC curves. The quality of these cutoff points was determined using the area under the ROC curve (AUC), with values ​​above 0.70 considered valid. Survival curves were estimated using the Kaplan-Meier method to analyze the probability of patient survival. Data were obtained on overall survival (OS) and event-free survival (EFS), considering events as relapse, progression, or death, and patients who had not experienced this event were censored at time of last follow up. Comparisons between groups were performed using the log-rank test.

## Results

### Patient characteristics

A total of 24 pediatric and young adult patients with R/R ALL received an infusion of tisagenlecleucel at our institution. Standardized CAR-T monitoring by MFC and overall outcomes were evaluated based on the use of BT with or without inotuzumab ozogamicin. Patient´s characteristics are described in **Table 1**. Ten patients (41,7%) received inotuzumab as BT, while 14 (58.3%) were classified as chemotherapy-steroids (chemo) based regimen. Among the 10 patients who received inotuzumab, 6 received it exclusively as BT after leukapheresis, whereas 4 had also received at least one Inotuzumab dose prior to leukapheresis. Three of these four patients received pre-apheresis inotuzumab due to logistical adjustments (leukapheresis rescheduling or need for a second leukapheresis after an out-of-specification product). The fourth patient had received inotuzumab 5 months before leukapheresis, prior to hematopoietic stem-cell transplantation. The baseline characteristics of patients in each treatment cohort were mostly well matched. Remarkably, no statistically significant differences were observed in the median blast percentage prior to BT 14.5% (95%CI 8.3%-54.7%) in the inotuzumab cohort vs 26% (95%CI 15-55.1%) in the chemotherapy cohort. Also, the median time of relapse after allogeneic hematopoietic stem cell transplantation (HSCT) was 6 months in the inotuzumab group as compared to 11 months in chemo BT patients (p=0.045). Nonetheless, patients receiving inotuzumab exhibited lower blast percentages and reduced bone marrow CD19^+^ B-cell precursor levels prior to infusion (post BT) compared with those in the chemotherapy cohort (p=0.017 and p=0.03, respectively) (**Table 1**).

**Table 1:** Baseline patients and disease characteristics prior and post BT.

Characteristics	Inotuzumab group N = 10	Chemo group N = 14	Total N = 24	p
Median age, years (range)	21 (2–24)	10 (2–25)	12.5 (2–25)	0.26
Female, n (%)	5 (50)	4 (28.5)	9 (37.5)	0.26
Citogenetic analyses:				
ETV6::RUNX1	0	1	1	
High Hyperdiploidy	0	1	1	
t (1,19) *additional p53 mutated	2 (*1)	0	2	
KMT2A gene fusions	1	0	1	
Low hypodiplody	1	0	1	
iAMP21	0	1	1	
IKZF1 deletion/mutation/Ikaros plus	2	2	4	
BCR-ABL	1	0	1	
B-other	1	7	8	
Non available	2	2	4	
Previous Allogeneic HSCT, n (%)	8 (80)	8 (57)	16 (66.7)	0.234
Previous blinatumomab, n (%)	2 (20)	0	2 (8.3)	0.163
Relapse after Allogeneic HSCT (months), median (range)	6 (3.2-9.1)	11 (6.6-19.7)	7.5 (5.7-11.7)	0.045
Median % bone marrow disease prior to BT (95% CI)	14.8 (8.3-54.7)	26 (15-55.1)	25 (19.6-47.6)	0.85
EM disease prior to BT, n (%)	2 (20)	2 (14.3)	4 (16.6)	1
Median % bone marrow disease post BT (pre-CAR-T cell infusion) (95% CI)	0 (0-0.45)	6.3 (0.07-29)	0.17 (0-6.3)	0.017
Median % baseline bone marrow B-cells post BT (pre-CAR-T cell infusion) (95% CI)	0.001 (0-4.3)	0.7 (0.1-4.9)	0.12 (0.08-2.2)	0.03
Median B-lymphocytes per μl peripheral blood post BT and pre-CAR-T cell infusion (95% CI)	5 (0–235)	215 (4–348)	11 (3–275)	0.144
Tumor burden dynamics: change in tumor burden after pre and post BT, n (%)				
Low to low tumor burden	0	4 (28.6)	4 (16.6)	0.032
High to low tumor burden	8 (80)	4 (28.6)	12 (50)	
High to high tumor burden	2 (20)	6 (42.8)	8 (33.3)	
Undetectable MRD pre-LD, n (%)	6 (60)	2 (14.3)	8 (33.3)	0.03
Complete blood cell counts prior to LD; median				
ANC, cells x 10^9^/L (95% CI)	0.9 (0.49-1.7)	0.79 (0.25-2.02)	0.82 (0.51-1.32)	0.81
Platelet count, x 10^9^/L (95% CI)	65.5 (37–86)	100 (62–168)	80.5 (61–131)	0.64
Hemoglobin, g/dL (95% CI)	10.6 (8.5-12.2)	10.8 (9.8-12.1)	10.7 (10.1-11.1)	0.7
Lactate dehydrogenase (mg/L) prior to LD, median (95% CI)	299 (222–354)	209 (189–248)	236 (204–299)	0.16
C-reactive protein prior to LD, median mg/dl (95% CI)	0.93 (0.21-2.25)	0.41 (0.1-1.7)	0.66 (0.21-1.5)	0.78
ALL Hematotox score (Nair M et al), n (%)				
Low-risk < 4 points	7 (70)	10 (71)	17 (71)	1
High risk ≥ 4 points	3 (30)	4 (29)	7 (29)	
ALL Hematotox score (modified), n (%)				
Low-risk ≤2 points	2 (20)	5 (35.7)	7 (29.2)	0.856
Intermediate 3–4 points	5 (50)	5 (35.7)	10 (41.7)	
High risk > 4 points	3 (30)	4 (28.6)	7 (29.2)	

LD, Lymphodepletion; BT, bridging therapy; HSCT, hematopoietic stem cell transplant; BM, Bone marrow; ANC, absolute neutrophil count; EM, extramedullary disease; CI, confidence interval; MRD, Mesurable residual disease.

### Overall and *event free survival*

**Figure 1:**
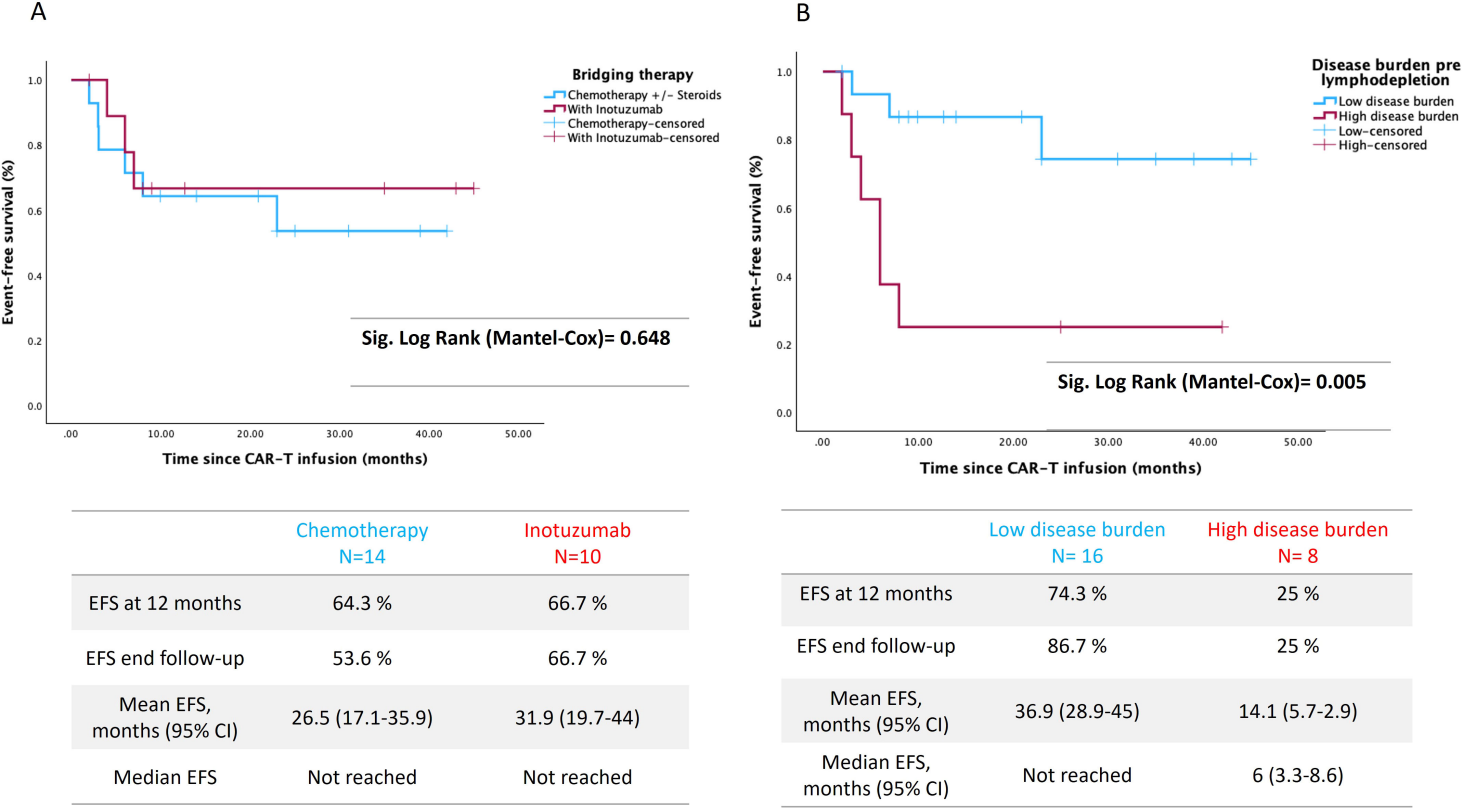
Event free survival: **(A)** Event free survival (EFS) depending on bridging therapy: inotuzumab versus chemotherapy +/- steroids. **(B)** EFS among patients with high tumor burden (HTB) vs low tumor burden (LTB).

**Figure 2:**
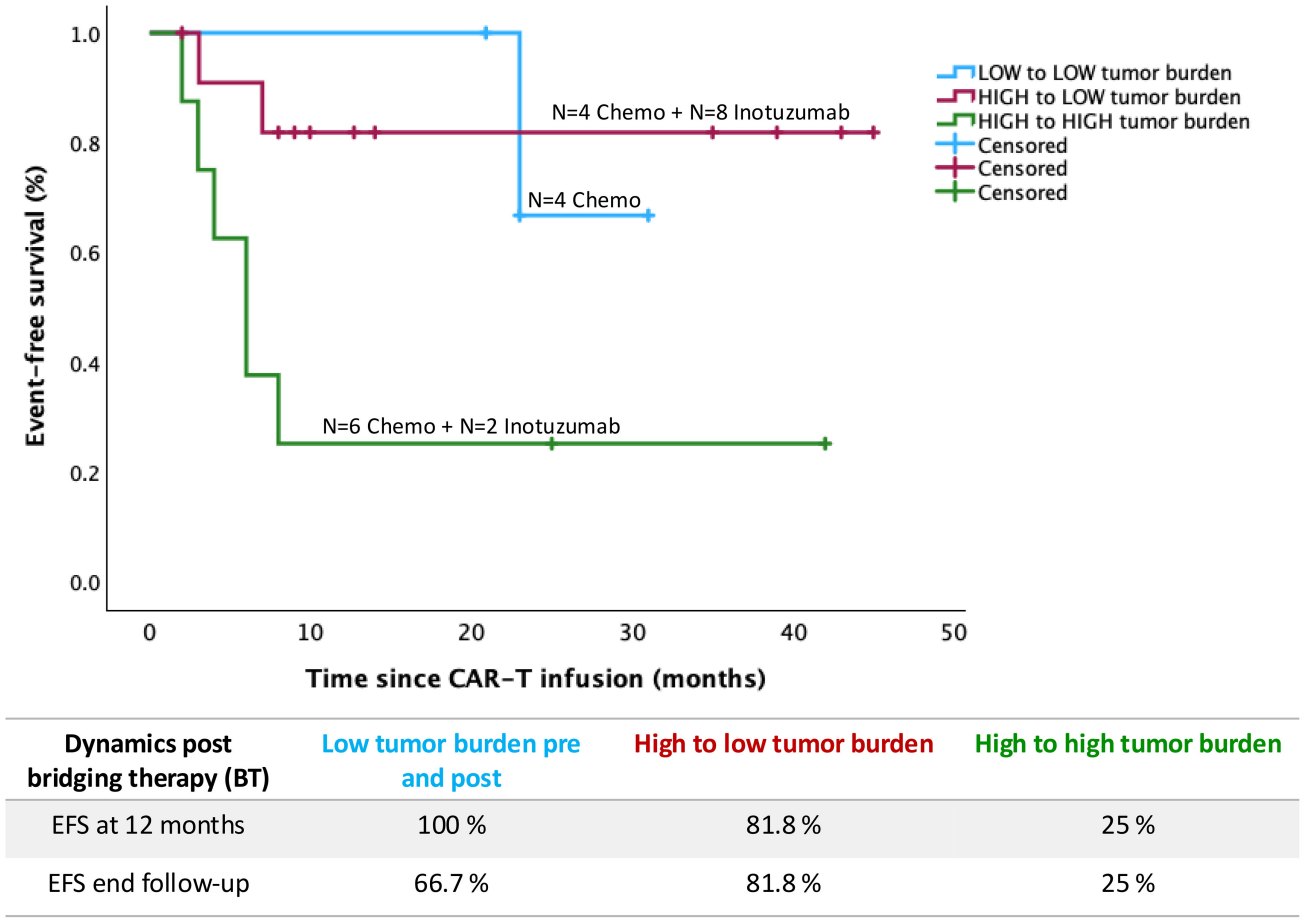
Event free survival (EFS) according to tumor burden dynamics through bridging therapy.

The EFS and OS at 12 months of the whole series of patients was 65.8% and 75.6%, respectively, with a median follow-up of 17 months (95% CI 11.4-23.7). There were no significant differences between the EFS of patients receiving either chemo or inotuzumab as BT, the 12-month EFS being 64.3 vs 66.7% respectively (p = 0.648) (**Figure 1A**). The mean EFS in the chemo group was 26.5 months (95% CI 17.1–35.9) compared to 31.9 months (95% CI 19.7–44) in the inotuzumab group. In contrast, leukemic burden had a significant impact on EFS. More specifically, EFS at 12-month was 74.3% vs 25% for patients with LTB and HTB, respectively, p=0.005 (**Figure 1B**). Remarkably, 8 of 10 patients (80%) with HTB before BT achieve LTB before LD in the inotuzumab group compared to only 4 patients (28.6%) in the chemo group (p=0.032). In fact, pre-LD negative MRD was achieved in 60% in the inotuzumab group compared to 14.3% in the chemo group (p=0.03) (**Table 1**). Moreover, regardless of BT, achieving or maintaining LTB gives rise to significantly improved EFS (p=0.019). Thus, EFS at 12-months was 81.8% and 100% respectively compared to 25% for those patients who maintained HTB (6 patients from chemotherapy group and 2 patients from inotuzumab as BT) (**Figure 2**).

### Toxicity and *response*

**Figure 3:**
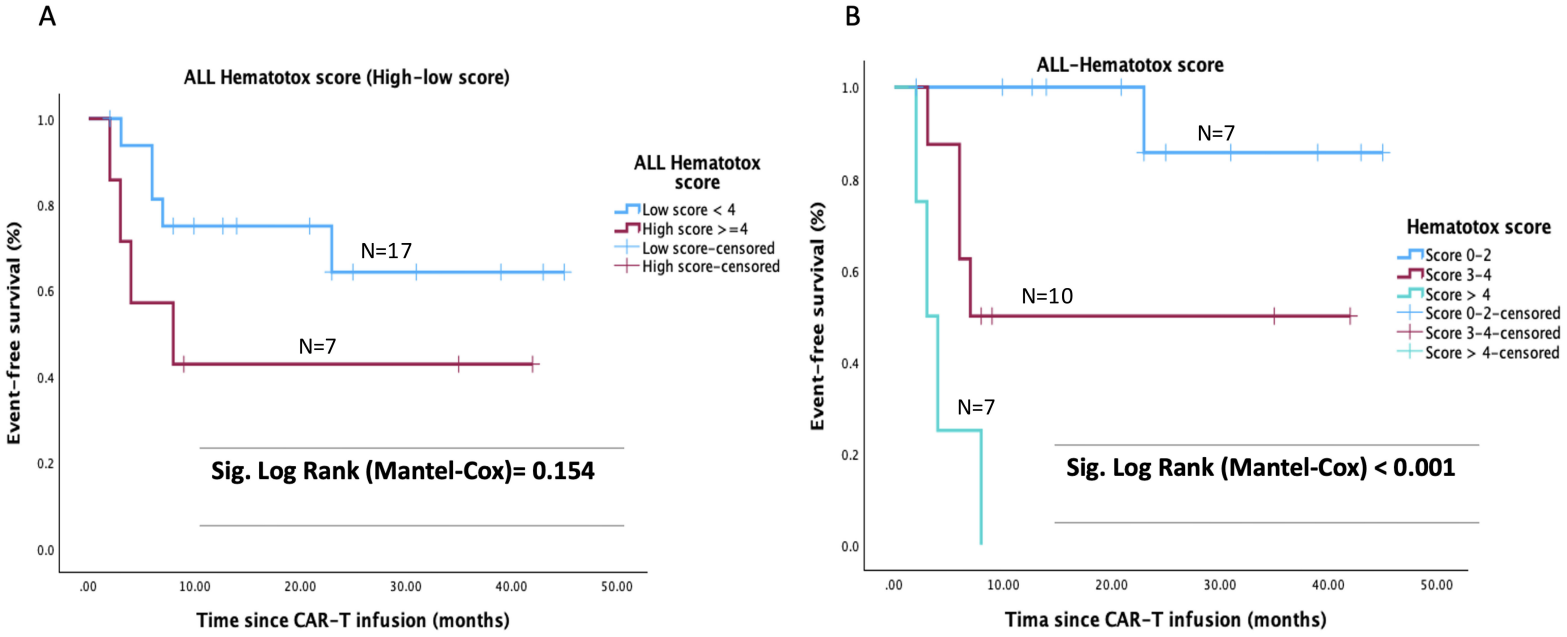
Event free survival (EFS) depending on Hematotox score: **(A)** high (≥4) vs low (<4) score definition and **(B)** score ≤ 2(low), 3-4 (intermediate) or > 4 (high).

The number of patients with severe cytokine release syndrome (CRS) was similar in both groups. In addition, all patients achieved CR/CRi on day 28 after CAR-T cell infusion with absolute BCA being observed in all of them. “The median duration of BCA after infusion was 3.8 months (range 0.5–2.7) among patients who received inotuzumab, vs. 6 months (range 3.4–8.5) in the chemotherapy group (p = 0.29). As detailed in **Table 2**, we distinguished whether BCA was lost due to early B-cell recovery in remission, relapse (including CD19-negative relapse), or consolidative HSCT. Despite the shorter global duration of BCA in the inotuzumab subgroup, the proportion of patients who maintained persistent BCA while remaining in remission without any competing event was comparable between groups (30% vs. 35.7% for inotuzumab and chemotherapy, respectively). Overall rates of BCA loss or competing events were also similar (70% vs. 64%). However, early recovery of BCA was more frequent in the inotuzumab group (60% vs. 35.7%), whereas CD19-negative relapses with preserved BCA occurred exclusively in the chemotherapy bridging subgroup (28.6%) (**Table 2**). Differences in patients’ EFS were also observed according to the pre-LD Hematotox score (**Figure 3A**). The 12-month EFS for patients with an Hematotox score ≤2 (low), score 3-4 (intermediate) or > 4 (high) was 100%, 50% and 0% respectively (p<0.001) (**Figure 3B**).

**Table 2:** Characteristics of CAR T-cell therapy: grading and management of toxicities, response at day 28 after CAR-T cell infusion and B-cell aplasia.

Characteristics	Inotuzumab group N = 10	Chemo group N = 14	Number of patients (N = 24)	p
Toxicity after infusion, n (%)				
- CRS any grade	9 (90)	14 (100)	23 (95.8)	0.194
CRS grade 1	7 (70)	7 (50)	14 (58.3)	
CRS grade 2	1 (10)	6 (43)	7 (29.2)	
CRS grade ≥ 3	1 (10)	1 (7)	2 (8.3)	0.6
- Immune effector cell-associated HLH-like syndrome	1 (10)	2 (14.3)	3 (12.5)	
- ICANS, n (%)	1 (10)	2 (14.3)	3 (12.5)	
Anti-cytokine therapy, n (%)				
Tocilizumab	3 (30)	9 (64.3)	12 (50)	0.10
Corticosteroids	1 (10)	2 (14.3)	3 (12.5)	0.62
Anakinra/Siltuximab	1 (10)	1 (7.1)	2 (8.3)	0.67
Disease response D28				
CR, n(%)	2 (20)	3 (21)	5 (21)	1
CRi*, n(%)	8 (80)	11 (79)	19 (79)	
MRD negativity D28, n (%)	10 (100)	14 (100)	24 (100)	
Relapse after CAR-T, n(%)	3 (30)	6 (42.9)	9 (37.5)	0.418
B-cell aplasia (BCA) follow-up:				
Persistent BCA	3 (30)	5 (35.7)	8 (33.3)	0.65
Loss of BCA (B-cell recovery or event (relapse or consolidative HSCT), n (%):	7 (70)	9 (64)	16 (66.7)	1
—CD19 negative relapse during BCA	0	4 (28.6)	4 (16.7)	0.11
—Consolidation HSCT during BCA	1 (10)	0	1 (4)	0.4
—B-cell recovery while in remission**	6 (60)	5 (35.7)	11 (46)	0.22
Median BCA duration (months, IC 95%) ***	3.8 (0.5-2.7)	6 (3.4-8.5)	4.6 (1.3-1.9)	
Event free BCA persistence	30%	35.7%	33.3%	0.62

*CRi: Complete remission with incomplete hematologic recovery was defined by CR but ≥ 1 of the following: neutrophils ≤ 1.0 x 10^9^/L, platelets ≤ 100 x 10^9^/L, or platelet/blood transfusions within 7 days.

**Definition of B-cell recovery: Reappearance of B-lymphocytes in peripheral blood after absolute B cell aplasia confirming an increase in count over time.

***BCA duration: Persistence B-cell aplasia until event: B-cell recovery, relapse/not response or HSCT.

### CAR-T cell expansion according to BT and burden tumor

**Figure 4:**
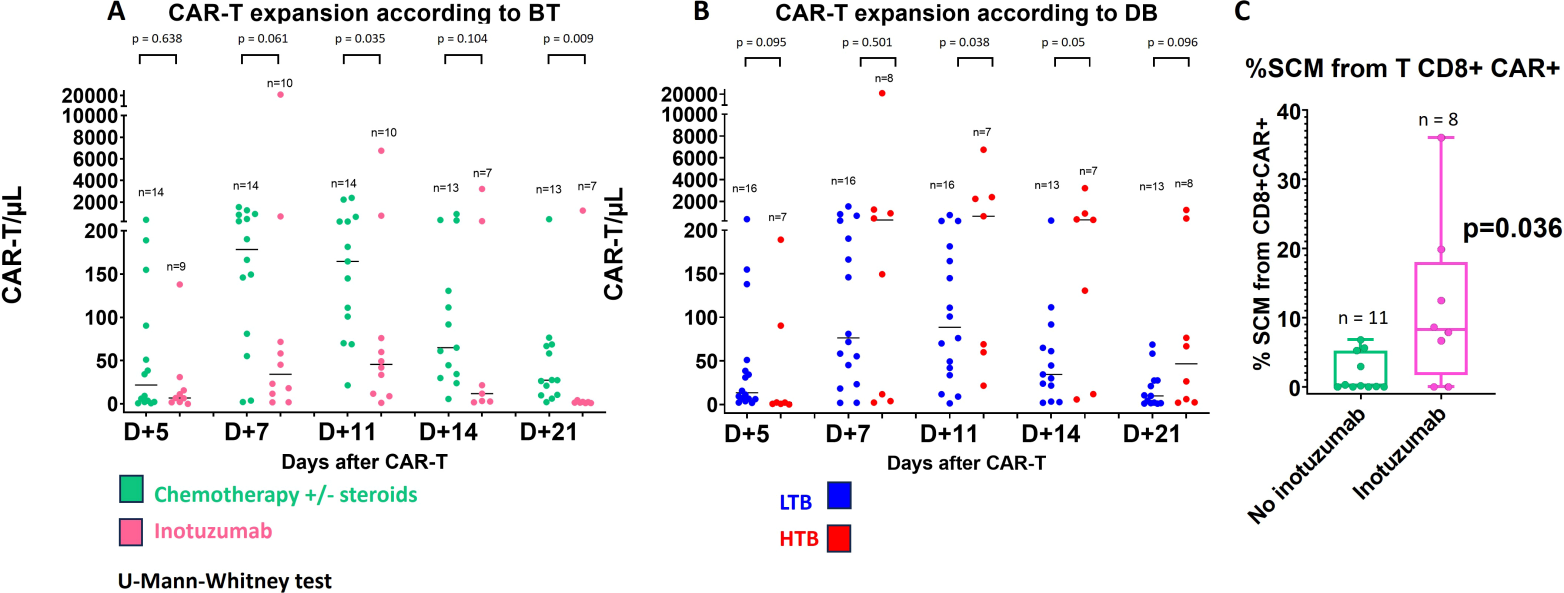
Characteristics of CAR-T cells: **(A)** CAR-T cell expansion according to bridging therapy and **(B)** % CAR-T cell expansion according to disease burden and **(C)** % of SCM according to bridging therapy. Statistical differences between groups were assessed using Mann-Whitney test. DB: Disease burden, HTB: high tumor burden, LTB: low tumor burden, SCM: Stem cell memory.

Concerning CAR-T monitoring, the median day of peak expansion of CAR-T cells was day +7 (range of 7–14 days) reaching a median of 190.4 cells/µL (range 3.27-20.889) and 24,2% of CD3+ T-cells (range 2.9-21-2). All but one patient in the inotuzumab group exhibited more than 5 CAR-T/µL after infusion and the same expansion kinetics previously described (25). However, the percentage and CAR-T/µL at the time of peak expansion were significantly different depending on BT. Accordingly, patients who receive inotuzumab as BT showed a significantly lower expansion: 10.2% of CD3+ T-cells (range of 2.85-85.1), 58.3 CAR-T/µL (range of 3.27-20.889.1), and AUC14 156.7 (range of 4.59-54471) compared to 30% (range of 7.3-91.2), 337.7 CAR-T/µL (range of 100.9-2.392.7) and AUC 467.4 (166.2-7.349) in the chemotherapy group (p=0.02, p=0.011 and p=0.15 respectively) (**Figure 4A**). Similarly, tumor burden influenced the expansion: patients in HTB had higher expansion of CAR-T cells, 50.78% of CD3+ T cells (range of 16.82 – 91.2), 771.65 CAR-T/µL (range of 59.2 – 20889) and AUC14 5699 (427 – 54471), compared to 11.34% of CD3+ cells (range of 2.85 – 59.8), 145 CAR-T/µL (range of 3.27 – 1537) and AUC14 330.7 (range of 4.6 – 4349.3) in LTB (p = 0.008, p = 0.017 and p = 0.009, respectively) (**Figure 4B**). Interestingly, among patients with LTB (16 patients), 8 (50%) received inotuzumab and also exhibited a significantly reduce expansion: 7.79% of CD3+ T-cells (range of 2.85-33.16), 49.3 CAR-T/µL (range of 3.27-681.7) and AUC14 136.5 (range of 4.6-1.419.3) in the inotuzumab group vs 25.07% of CD3+ T-cells (range of 7.3-59.9), 197.5 CAR-T/µL (100.9-1.537.1) and AUC14 413.79 (166.2-4349.3) in the chemo group, (p=0.029, p=0.009 and p=0.065) respectively (**Table 3**). No differences in long-term CAR persistence were observed (**Supplementary Figure 5**). In **Supplementary Table 5**, CAR-T cell expansion is correlated with tumor burden and residual B- cell population prior to CAR therapy and post-BT.

**Table 3:** Biological characteristics of CAR T-cell therapy according to BT.

Characteristics	Inotuzumab group N = 10	Chemo group N = 14	Total N = 24	p
Expansion > 5 CAR-T/µL Yes n (%)	9	14	23	
Day max peak	7 (7–14)	7 (7—11)	7 (7–14)	0.829
% CAR from CD3+	10.2 (2.85-85.1)	30 (7.3-91.2)	24.2 (2.9-91.2)	**0.02**
CAR-T/µL	58.3 (3.27-20.889.1)	337.7 (100.9-2.392.7)	190.4 (3.27-20.889)	**0.011**
AUC_14	156.7 (4.59-54471)	467.4 (166.2-7.349)	425.2 (4.59-54.471)	0.15
Low disease Burden				
N (%)	8	8	16	
% CAR from CD3+	7.79 (2.85-33.16)	25.07 (7.3-59.9)	11.34 (2.85-59.8)	**0.029**
CAR-T/µL	49.3 (3.27-681.7)	197.5 (100.9-1.537.1)	145 (3.27-1537.4)	**0.009**
AUC_14	136.5 (4.6-1.419.3)	413.79 (166.2-4349.3)	330.7 (4.6-4349.3)	0.065
Characteristics of expansion (N studies)	9	13	22	
Exhaustion markers (% from CD8)				
PD1	69.72 (32 - 96.41)	87.81 (44.64-97.18)	76.79 (32-97.18)	0.442
LAG3	26.4 (16 - 38.14)	26.97 (5.52 - 93.99)	26.97 (5.52-93.99)	0.734
Ki67	83.44 (26.08 - 100)	97.63 (32.14 - 100)	95.29 (26.08 - 100)	0.24
Subpopulation CAR-T (% from CD8)				
*Naïve* (%)	8.24 (0 - 40.44)	0.58 (0 - 6.81)	4.64 (0 - 40.44)	0.075
*SCM (%)	8.24 (0 - 36.03)	0.14 (0 - 6.81)	2.93 (0 - 36.03)	**0.036**
*Central memory* (%)	49 (17.54 - 73.33)	62.45 (0 - 86.2)	61.76 (0 - 86.2)	0.364
*SCML (%)	1.4395 (0 – 10)	0 (0 - 3.27)	0 (0 – 10)	0.326
*Peripheral memory* (%)	32.34 (6.14 - 68.42)	26.6 (0 - 90.19)	26.61 (0 - 90.19)	0.509
*Effector* (%)	0.37 (0 - 14.04)	0.19 (0 - 6.63)	0.19 (0 - 14.04)	0.864
*Immature effector (%)	0 (0 - 3.51)	0 (0 - 3.93)	0 (0 - 3.93)	1
*Intermediate effector (%)	0.37 (0 - 8.77)	0.19 (0 - 5.61)	0.19 (0 - 8.77)	0.864
*Final effector (%)	0 (0 - 1.75)	0 (0 - 0.26)	0 (0 - 1.75)	0.965

SCM, Stem cell memory; SCML, Stem cell memory like; AUC, Area under curve; mDC, myeloid dendritic cells.

Statistically significant data is highlighted in bold.

Immunophenotyping of CAR-T cells was performed at the time of peak of expansion, and no significant differences were observed between both subgroups in terms of proliferation (ki67), activation (CD71) or exhaustion markers (PD1, LAG3) (**Supplementary Table 2**). However, the analysis of T-cell subpopulations revealed a higher proportion of SCM T-cells within the CD8+ CAR-T cells of patients from the inotuzumab group: median 8.24% (range 0 – 36.03) vs 0.14 (0 – 6.81) in chemo group, p= 0.036 (**Figure 4C**). On the other hand, when we consider the tumor burden, a greater proliferation of CD4+ CAR-T cells was observed: Ki67 99.46% of CD4+ CAR-T (range 72.12 – 100) in HTB vs 80.64 (13.61 – 100) in LTB group, p = 0.03.

### Leukapheresis according to BT and burden tumor

The different lymphocyte populations, including CD8+ SCM and stem cell memory like cells (SCML), as well as the rest of the hematopoietic cells collected during apheresis, were studied by MFC. Considering tumor burden, we detected a higher percentage of total monocytes and specifically more intermediate and fewer non-classical ones, a higher percentage of plasmacytoid and mDC and lower percentage of lymphoblasts in patients with LTB vs HTB: 25.8% (range of 4.35% – 41.57%) vs 2.85% (range 0 – 0.04), p = 0.002; 0.18% (range 0-0.75) and 0.41% (range 0.09 – 1.73) vs 0.01% (range 0 – 0.04) and 0.02% (0 – 0.018), p = 0.008 and p = 0.001; and 0% (range 0- 0.24) vs 7.97% (range 0 – 54.98), p = 0.002, respectively (**Supplementary Figure 6**). Also, a trend toward a higher percentage of SCML cells among CD8+ cells was observed in those patients with LTB: 1.08% (0.33 – 3.71) vs 0.55% (0.19 – 1.05), p = 0.07).

### Immune reconstitution after CAR-T cell therapy

**Figure 5:**
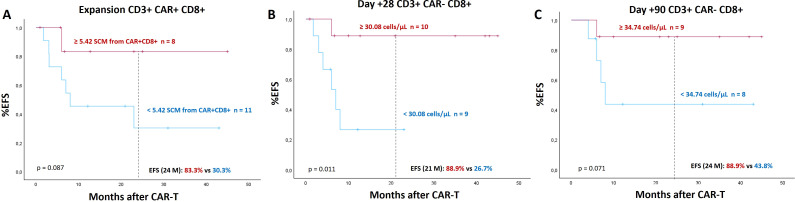
Prognostic value of CD8+ stem cell memory (SCM) cells. **(A)** EFS according to percentage of SCM cells among CD8+ CAR-T cells at the time of peak of expansion, **(B)** EFS according to CD8+CARneg SCM/µL 28 days after CAR-T cell infusion and **(C)** after 90 days after CAR-T cell infusion. Cut-off points were determined using ROC curves or median values, and statistical differences between groups were assessed using log-rank test. Differences are considered significant (p<0.05) and shown in bold. SCM: Stem cell memory. EFS: Event free survival.

Regarding the immune populations and CAR-T cells identified by MFC on day +28, no differences were observed according to BT or tumor burden. However, if we consider the patients who eventually relapsed during the follow-up period, it was observed that patients who remain in CR had a higher percentage of *naive* and SCM cells among CD8+ CAR-T cells: 16.7% (10.61 – 46.67) and 10.61 (0 – 33.33) vs 0% (0 – 24.61) and 0% (0 – 0), p = 0.041 and p = 0.019 (**Supplementary Tables 3, 4**). Based on these results, we aimed to evaluate the EFS of patients based on the SCM CD8+ CAR-T pos or CAR-T neg cell populations. Thus, we observed a shorter EFS among those patients who presented a lower percentage of SCM cells among CD8+ CAR-T cells at the peak of expansion (**Figure 5A**) and lower SCM CD8+CARneg/µL on 28 days (**Figure 5B**) and on 90 days after CAR-T therapy (**Figure 5C**).

## Discussion

The integration of immunotherapeutic agents such as inotuzumab ozogamicin into salvage regimens for R/R ALL has significantly reshaped the therapeutic landscape, particularly in the context of bridging strategies prior to CD19-directed CAR-T cell therapy. This paradigm shift raises critical questions regarding the optimal sequencing of treatments, the potential impact of inotuzumab on CAR-T efficacy and expansion, and how best to balance toxicity with therapeutic benefit—especially considering factors like pre-infusion BCA and tumor burden (11, 25).

In this study, we present a retrospective analysis of 24 patients treated with tisagenlecleucel, of which fourteen received conventional chemotherapy as BT, while ten received inotuzumab. While overall outcomes between the two groups appeared similar, our analysis is, to our knowledge, the first to examine how inotuzumab, compared to chemotherapy, modifies pre-infusion tumor burden and influences the characteristics and kinetics of CAR-T cell expansion measured by MFC.

Previous studies performed on pediatric population have reported conflicting results regarding the impact of inotuzumab prior to CAR-T cell infusion. Mullanfiroze et al. observed suboptimal outcomes and significantly lower EFS in patients receiving inotuzumab prior to CAR-T cell infusion (12%) compared to those who did not (53%, *p<*0.001) (26). In addition, other data raise concerns about the potential negative effects of inotuzumab on CAR-T cell expansion. Krueger et al. and Dourthe et al. reported impaired CAR-T cell expansion and poorer overall survival (OS) in patients who had received inotuzumab as BT (15, 27). Moreover, Lust et al. identified inotuzumab as an independent factor associated with a worse OS and relapse-free survival (RFS) in a real-world cohort of young adult patients, with a hazard ratio (HR) of 6.32 (95% CI, 1.48–27) (28). They also noted that patients with LTB at infusion had better OS than those with HTB yet did not systematically assess post-bridging tumor burden status.

A key limitation in many of these studies is that they included patients exposed to inotuzumab both before apheresis and as BT, which may introduce confounding factors linked to disease biology—i.e., patients receiving inotuzumab may have had a more aggressive disease. Supporting this, Aldoss et al. demonstrated that while prior inotuzumab exposure was associated with inferior outcomes after brexucabtagene autoleucel in unadjusted analyses, it lost statistical significance in multivariate analysis and did not impact progression-free survival (PFS) (HR 1.20; 95% CI, 0.71–2.03) (29). Notably, responders to inotuzumab showed superior outcomes, suggesting efficacy of prior therapy may be more relevant than exposure per se.

The role of inotuzumab in modulating pre-infusion BCA and its effect on CAR-T cell expansion is still not well understood. A recent report by Sahai et al. highlighted a high prevalence of pre-existing BCA (70.4%) among patients undergoing CAR-T cell therapy (30). Interestingly, the frequency of prior inotuzumab was similar in patients with or without pre-existing BCA (17.5% vs. 20.8%, *p* = 0.66), suggesting inotuzumab may not be a dominant contributor to BCA.

In our cohort, patients in the inotuzumab group exhibited significantly lower levels of circulating B-lymphocytes and bone marrow B-cell progenitors prior to infusion compared to those receiving chemotherapy-based BT. While CAR-T cell expansion was adequate in nearly all patients, its magnitude was reduced in the inotuzumab group irrespective of the tumor burden. These findings support the hypothesis that inotuzumab contributes to reducing antigenic exposure through B-lymphopenia, which may dampen CAR-T cell expansion without necessarily compromising efficacy. Indeed, despite a reduced expansion, we were able to detect an enrichment in SCM CD8+ T-cells in the inotuzumab group. These lymphocytes have self-renewal capacity, can differentiate into others effector T-cell subpopulations and have long-term persistence potential (31). Consistent with other studies, we have observed that a higher presence of these cells correlates with a better prognosis (32, 33). The use of inotuzumab as BT proved a high effectiveness in reducing tumor burden and the residual CD19+ B-cell population. This finding suggests a significantly reduced antigen exposure for the subsequently infused CAR-T cells. Crucially, the quality of CAR-T cell expansion was not negatively affected, as evidenced by the preserved T-cell subset distribution and the stable expression of senescence molecules. This lack of impact is consistent with its mechanism of action: inotuzumab targets CD22 directly and, unlike bi-specific T-cell engagers, does not involve T lymphocytes in its cytotoxic activity. Our outcomes align with recent studies showing that inotuzumab as a bridging agent does not adversely affect outcomes. Ceolin et al. reported no significant differences in outcomes following inotuzumab prior to CAR-T cell therapy in a pediatric cohort (34), and Rubinstein et al. similarly found comparable response rates between inotuzumab and chemotherapy-based BT in a retrospective pediatric study (16). However, neither study provided detailed data on the quality of CAR-T cell expansion or post-bridging tumor burden.

Importantly, our findings underscore the ability of inotuzumab to effectively reduce pre-infusion tumor burden. In our cohort, 80% of patients in the inotuzumab group converted from high to low tumor burden after BT, compared to only 40% in the chemotherapy group. As shown in multiple studies, high pre-infusion tumor burden is a major predictor of poor outcomes following CAR-T cell therapy (10, 35–37). In fact, tumor burden has been incorporated into the recently validated ALL-Hematotox score, where marrow involvement >5% or >25% adds 1 or 2 points, respectively, correlating with both hematologic toxicity and survival (24). In our series, the Hematotox score significantly stratified EFS, with 100%, 50%, and 0% EFS in patients with scores of 0–2, 3–4, and >4, respectively (p<0.001). Moreover, ALL-Hematotox score categories were balanced across bridging strategies, with only a minor, non-significant trend toward fewer low-risk patients in the Inotuzumab group. As the score integrates factors beyond tumor burden—including bone marrow reserve and inflammatory markers, no meaningful imbalance was detected. Therefore, hematotoxicity risk is unlikely to have introduced selection bias or confounded the EFS comparison.

This study has several limitations. The use of inotuzumab as BT was not randomized and reflected individualized real-world decision-making, introducing potential selection bias. In addition, the small sample size limits the ability to perform comprehensive multivariable analyses, particularly regarding baseline disease biology, previous HSCT and timing of post-allo-HSCT relapses. Despite these limitations, our study provides strengths not jointly addressed in previous reports. By evaluating inotuzumab strictly within the context of BT choice, we offer a more accurate assessment of its impact on pre-infusion tumor burden, a parameter closely linked to event-free survival, and we complement this with detailed profiling of CAR-T phenotype, expansion, and *in vivo* behavior through prospective MFC monitoring and comprehensive immune subtyping that may contribute to a better understanding of the impact of inotuzumab as a bridging strategy. The integration of these two dimensions adds clinical relevance, as BT remains non-standardized and highly individualized in current practice.

In summary, while inotuzumab-based BT may be associated with reduced CAR-T expansion —potentially via B-cell aplasia— our data suggest this is outweighed by its superior capacity to decrease tumor burden prior to infusion. As high tumor burden is a dominant driver of relapse and toxicity, the net effect of inotuzumab may be favorable in appropriately selected patients. Prospective trials evaluating CAR-T cell kinetics, immune monitoring, and tumor dynamics after various bridging strategies are warranted to refine treatment sequencing and optimize outcomes.

## Data Availability

The raw data supporting the conclusions of this article will be made available by the authors, without undue reservation.
