# Supplementary material for: Impact of inotuzumab ozogamicin as bridging therapy and tumor burden in CAR-T therapy for B-acute lymphoblastic leukemia
Source: Front Immunol. 2025 Dec 18;16:1725878. doi: 10.3389/fimmu.2025.1725878 (PMC12756125; doi:10.3389/fimmu.2025.1725878)
Supplement: Supplementary file 1 [file DataSheet1.pdf]

Supplementary materials

1. **Supplementary table 1. Combination of antibodies and fluorochromes used for monitoring CAR-T cells and the immune system.** Page 2.
2. **Supplementary figure 1: Workflow diagram.** Page 3.
3. **Supplementary figure 2: Flow cytometry analysis of CAR-T cells.** Page 4.
4. **Supplementary figure 3: Flow cytometry analysis of T cells subpopulations.** Page 5.
5. **Supplementary figure 4: Flow cytometry analysis of activation and exhaustion biomarkers.** Page 6.
6. **Supplementary table 2: Exhaustion markers in CAR-T expansion according to BT. Page 7.**
7. **Supplementary table 3: T subpopulations of unmodified T lymphocytes and CAR-T cells analysed on day +28 post CAR-T and phenotype.** Page 8**.**
8. **Supplementary table 4. Supplementary table 3: NK cells, B lymphocytes and other subpopulations of immune system analysed on day +28 post CAR-T and phenotype.** Page 9.
9. **Supplementary figure 5: CAR-T cell persistence according to BT and disease burden.** Page 10.
10. **Supplementary table 5: CAR-T cell expansion correlated with tumor burden and residual B-cell population prior to CAR therapy and post-BT.** Page 11.
11. **Supplementary figure 6. Characteristics of apheresis**. Page 12.

Supplementary Table 1. Combination of antibodies and fluorochromes used for monitoring CAR-T cells and the immune system

Antibody	Clon	Company	Reference
**CD19 CAR detection reagent**		Miltenyi	130-129-550
**CD107a Pe-Cy7**	H4A3	BD	561348
**CD11c Red718**	B-ly6	BD	566932
**CD123 APC**	AC145	Miltenyi	130-113-322
**CD14 APC-H7**	MφP9	BD	641394
**CD16 Pe-Cy7**	3G8	BD	557744
**CD27 BV510**	O323	Biolegend	302836
**CD3 APC**	SK7	BD	345767
**CD3 APC-H7**	SK7	BD	641415
**CD3 perCPcy 5.5**	SK7	BD	332771
**CD3 Red718**	SP34-2	BD	566955
**CD4 APC**	SK3	BD	345771
**CD4 BV450**	RPA-T4	BD	560345
**CD4 BV605**	RPA-T	BD	562658
**CD45 BV605**	HI30	BD	564047
**CD45 V500**	HI30	BD	560777
**CD45RO Pe-Cy7**	UCHL1	BD	337168
**CD56 PE**	MY31	BD	345810
**CD62L FITC**	SK11	BD	347443
**CD71 APC-H7**	MA-712	BD	655408
**CD75RA Red718**	5H9	BD	752257
**CD8 APC-H7**	SK1	BD	641400
**CD8 FITC**	SK1	BD	345772
**CD8 Pe-Cy7**	SK1	BD	335822
**CD95 PE**	DX2	BD	555673
**HLADR BV711**	G46-6	BD	563696
**Ki67 PE**	RUO	BD	556027
**Lag3 APC**	7H2C65	BD	369211
**PD1 PerCP-Cy5.5**	EH12.1	BD	561273
**Streptavidin PE**	REA746	Biolegend	405203
**Biotin Vioblue**	REA746	Miltenyi	130-110-958
**FASL APC**	MFL3	Biolegend	106609

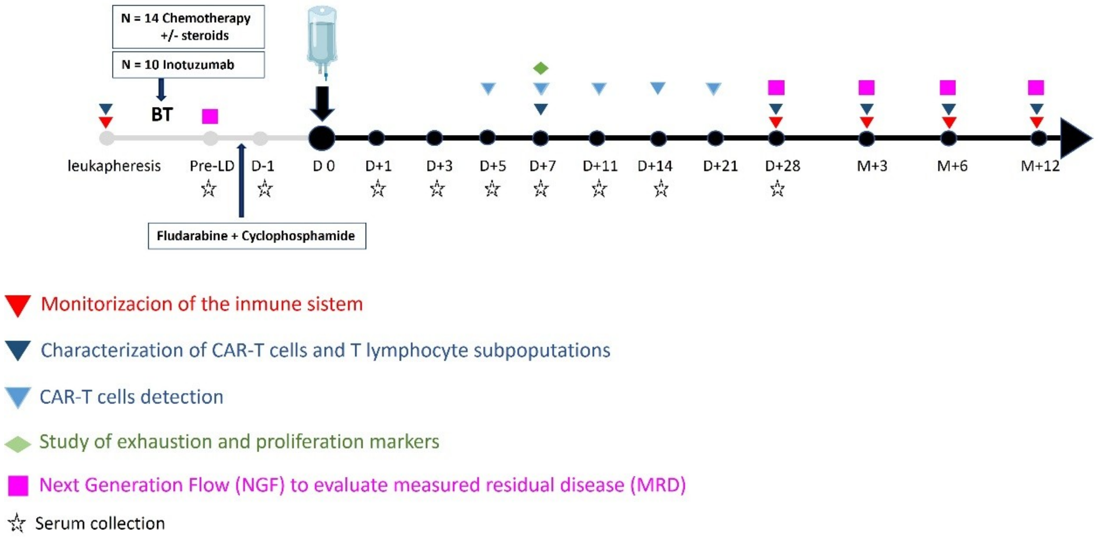

Supplementary figure 1: Workflow diagram. BT: bridging therapy. Pre-LD: Before lymphodepletion

.

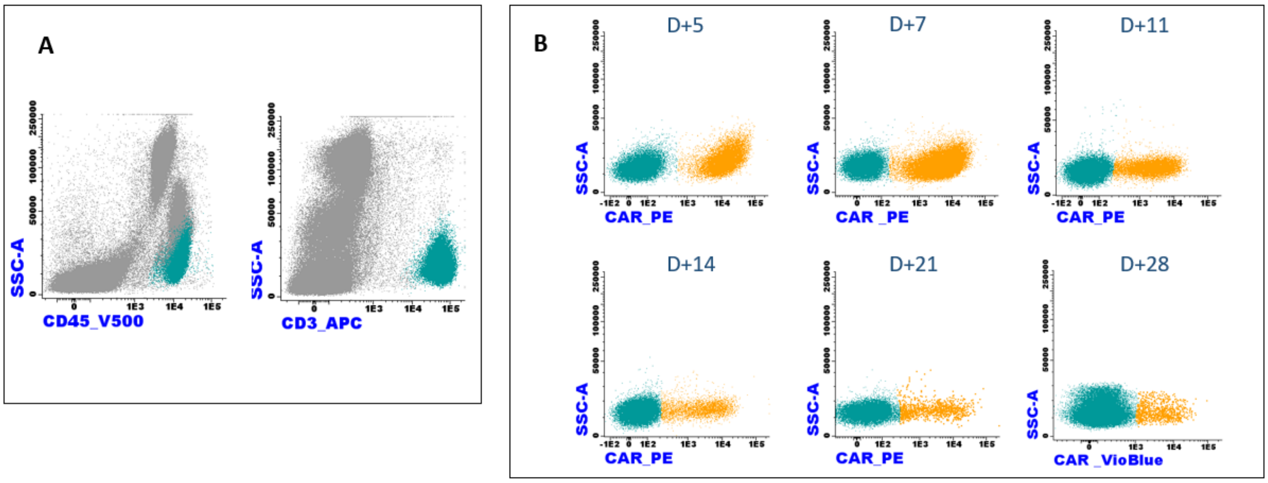

C

$$\text{AUC 14} = \frac{CART\ D7 - CART\ D5}{2(7-5)} + \frac{CART\ D11 - CART\ D7}{2(11-7)} + \frac{CART\ D11 - CART\ D11}{2(14-11)}$$

Supplementary figure 2: Flow cytometry analysis of CAR-T cells. (A) Identification of lymphocytes in aqua green based on the CD45 versus SSC plot, considering the least complex population (low SSC) and with bright CD45 expression. (B) Subsequently, we identify the CAR-positive population within the CD3+ T cells based on the expression of the CD19 protein using indirect immunofluorescence: CAR Detection Reagent (Miltenyi ref. 130-129-550) and Streptavidin-PE (Biolegend ref. 405203) or Biotin-VioBlue (Miltenyi ref 130-110-958). CAR-T/µL count was determined based on the percentage of CAR-T cells within the population and the same-day complete blood count (CBC). (C) The formula used for calculating the 14 (Area Under the Curve up to Day 14) for CAR-T cell expansion kinetics.

.

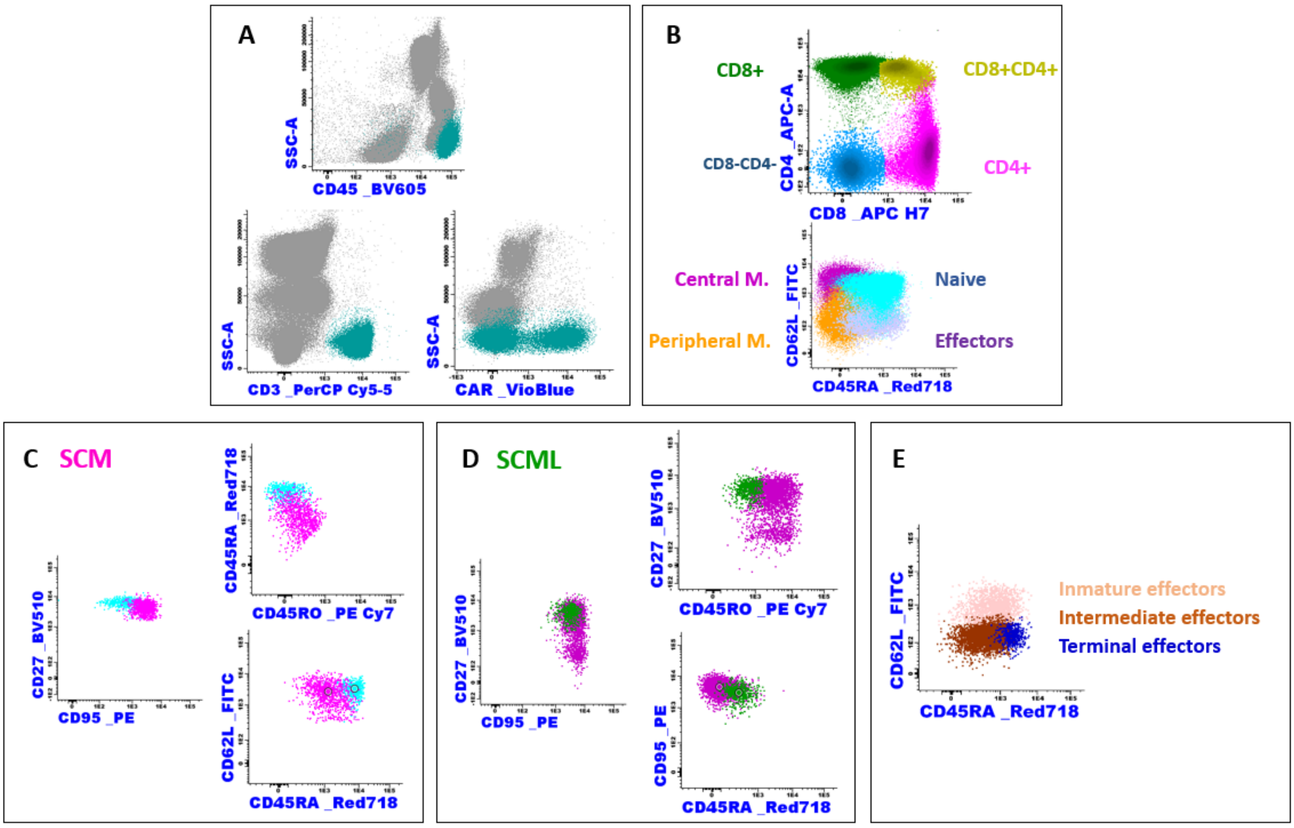

Supplementary figure 3: Flow cytometry analysis of T cells subpopulations. (A) Initial gating using CD45, CD3, and CAR stain. (B) Subpopulation of CAR-T cells based on CD4 and CD8 expression and differentiation of *naive,* central memory, peripheral memory and effectors according to the expression of CD62L and CD45RA. (C) Gating strategy for stem cell memory T cells (SCM). (D) Identification of stem cell memory like T cells (SCML). (E) Separation of effector T cells into immature (CD62L+), intermediate (CD62L- CD45RA+), and terminal subpopulations (CD62L- CD45RA++).

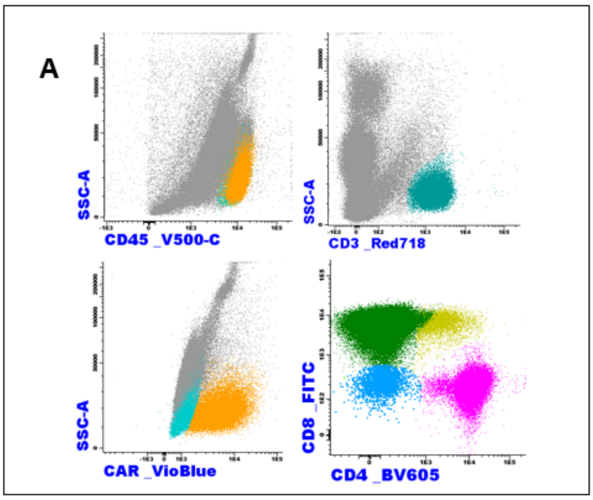

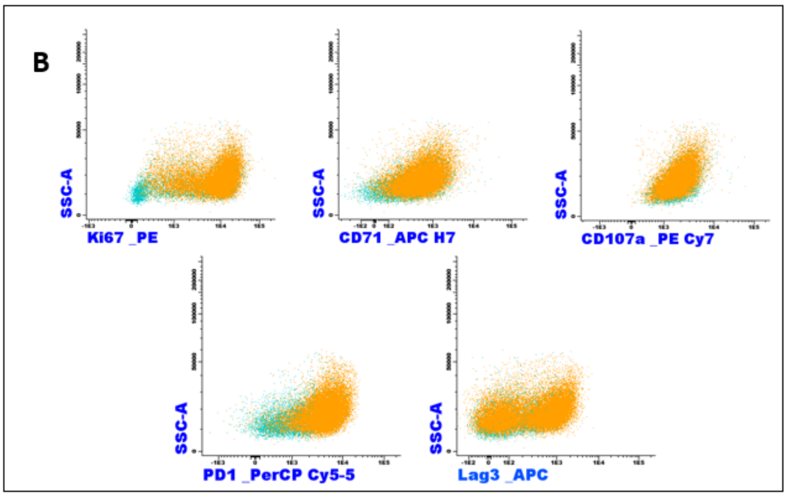

Supplementary figure 4: Flow cytometry analysis of activation and exhaustion biomarkers. (A) Initial gating using CD45, CD3, CAR, CD4 and CD8 markers. (B) Analysis of proliferation marker (Ki67), activation marker (CD71), degranulation marker (CD107a) and exhaustion markers (PD1 and LAG3).

Supplementary table 2: ***Exhaustion markers in CAR-T expansion according to BT***

% (range) from CD3+ CAR+	Global	Chemotherapy +/- steroids (n =11)	Inotuzumab (n = 8)	p value
% PD1 CD8 CAR	76.79 (32.05 - 97.18)	87.81 (44.64 - 97.18)	69.715 (32.05 - 96.41)	0.254
% LAG3 CD8 CAR	26.97 (5.52 - 93.99)	26.97 (5.52 - 93.99)	26.395 (16 - 38.14)	0.968
% PD1 LAG3 CD8 CAR	22.3 (1.52 - 80.8)	27.98 (1.52 - 80.8)	19.06 (6 - 34.83)	0.792
% PD1 CD4 CAR	78.75 (34.38 - 94.39)	82.47 (59.52 - 94.39)	63.48 (34.38 - 93.66)	0.099
% LAG3 CD4 CAR	18.945 (1.47 - 73.42)	20.475 (1.47 - 73.42)	11.97 (4.48 - 33.86)	0.537
% PD1 LAG3 CD4 CAR	11.35 (1.03 - 88.46)	18.6 (2.68 - 88.46)	10.785 (1.03 - 25.23)	0.364
% Ki67 CD8 CAR	95.29 (26.08 - 100)	97.63 (32.14 - 100)	83.44 (26.08 - 100)	0.243
% CD69 CD8 CAR	3.885 (0 - 20.54)	0.7 (0 - 8)	20.54 (20.54 - 20.54)	0.143
% CD71 CD8 CAR	1.985 (0 - 69.12)	2.61 (0.69 - 69.12)	0.5 (0 - 16.31)	0.291
% Treg CAR	8.635 (0 - 66.32)	2.43 (0 - 66.32)	14.84 (14.84 - 14.84)	0.655
% CD25 CD4 CAR	1.725 (0 - 19.73)	2.25 (1.2 - 19.73)	0 (0 - 0)	0.18
% Ki67 CD4 CAR	92.91 (13.61 - 100)	97.35 (35.57 - 100)	76.175 (13.61 - 100)	0.095
% CD69 CD4 CAR	5.695 (0 - 30.32)	5.58 (0 - 6.05)	30.32 (30.32 - 30.32)	0.143
% CD71 CD4 CAR	6.275 (0 - 26.94)	3.08 (0.69 - 21.99)	9.47 (0 - 26.94)	0.935

Supplementary table 3: T subpopulations of unmodified T lymphocytes and CAR-T cells analysed on day +28 post CAR-T and phenotype

Cell type at day +28 after CAR-T	Non-relapses after CAR-T N=13 Median (range)	Relapses after CAR-T N=7 Median (range)	P
**Unmodified T cell** (CD3+ CAR-) **cell/µL**	640,338 (79,62 - 3916,08)	595,2 (182,57 - 1107,04)	0,569
**UnmodifiedT CD8+** (CD3+, CD8+, CD4-) **cell/µL**	348,528 (33,57 - 2120,77)	259,072 (83,41 - 854,08)	0,97
% CD8 naive (CD3+, CAR-, CD8+, CD4-, CD45RA+, CD62L+, CD27++, CD95-)	15,852 (0 - 80,11)	10,3271 (1,22 - 38,52)	0,364
% CD8 Stem cell memory (CD3+, CAR-, CD8+, CD4-, CD45RA+, CD62L+, CD27++, CD95+)	11,68 (0-76,77)	8,99 (1,05-29,58)	0,393
% CD8 Stem cell memory like (CD3+, CAR-, CD8+, CD4-, CD45RAlow, CD62L+, CD27++, CD95low)	0,52 (0-2,57)	0,3 (0-2,2)	0,938
% CD8 Central memory (CD3+, CAR-, CD8+, CD4-, CD45RA-, CD62L+, CD27+/-, CD95+)	16,1876 (1,83 - 35,01)	22,3587 (2,44 - 54,16)	0,248
% CD8 Peripheral memory (CD3+, CAR-, CD8+, CD4-, CD45RA-, CD62L-, CD27+/-, CD95+)	31,4163 (2,97 - 57,8)	34,1206 (14,38 - 50,47)	0,804
% CD8 Inmadure effector (CD3+, CAR-, CD8+, CD4-, CD45RA+, CD62L+low, CD27+low, CD95+)	8,72 (0-11,67)	1,19 (0-20,15)	0,536
% CD8 Intermediate effector (CD3+, CAR-, CD8+, CD4-, CD45RA+, CD62L-, CD27+low, CD95+)	8,7 (1,12-20,22)	7,4 (o-28,24)	1
% CD8 Final effector (CD3+, CAR-, CD8+, CD4-, CD45RA++, CD62L-, CD27-, CD95+)	4,56 (1,38-33,3)	6,32 (0,39-25,46)	0,757
**Unmodified T CD4+** (CD3+, CD4+, CD8-) cell/µL	312,684 (32,49 - 1660,81)	255,717 (79,22 - 342,77)	0,053
% CD4 Naive (CD3+, CAR-, CD4+, CD8-, CD45RA+, CD62L+, CD27++, CD95-)	22,5041 (0,35 - 33,18)	7,4642 (1,57 - 47,31)	0,342
% CD4 Central memory (CD3+, CAR-, CD4+, CD8-, CD45RA-, CD62L+, CD27+/-, CD95+)	40,8578 (0 - 63,81)	34,4165 (18,14 - 90,39)	0,382
% CD4 Peripheral memory (CD3+, CAR-, CD4+, CD8-, CD45RA-, CD62L-, CD27+/-, CD95+)	38,0671 (6,88 - 83,41)	33,67 (3,2 - 78,82)	0,382
% CD4 Effector (CD3+, CAR-, CD4+, CD8-, CD45RA+, CD62L+, CD27-, CD95+)	1,1287 (0 - 11,55)	3,272 (0 - 9,11)	0,085
**Unmodified T CD8+CD4+** (CD3+, CAR-, CD8+, CD4+) **cell/µL**	11,375 (1,23 - 53,19)	5,476 (0,48 - 58,48)	0,063
**Unmodified T CD8-CD4-** (CD3+, CAR-, CD8-, CD4-) **cell/µL**	22,436 (4,78 - 87,32)	8,694 (0,49 - 44,78)	0,074
**CAR-T cell** (CD3+ CAR+) **cell/µL**	9,196 (0 - 73,79)	37,518 (0 - 548,08)	0,296
**CAR-T CD8+** (CD3+, CD8+, CD4-) **cell/µL**	7,092 (3,22 - 58,67)	39,7885 (0,48 - 461,72)	0,584
% CD8 naive (CD3+, CAR+, CD8+, CD4-, CD45RA+, CD62L+, CD27++, CD95-)	16.7 (10.61 - 46.67)	0 (0 - 24,61)	**0,041**
% CD8 Stem cell memory (CD3+, CAR+, CD8+, CD4-, CD45RA+, CD62L+, CD27++, CD95+)	10.61 (0 - 33.33)	0 (0 - 0)	**0.019**
% CD8 Stem cell memory like (CD3+, CAR+, CD8+, CD4-, CD45RAlow, CD62L+, CD27++, CD95low)	0 (0 - 6,67)	0 (0 - 0,74)	0,368
% CD8 Central memory (CD3+, CAR+, CD8+, CD4-, CD45RA-, CD62L+, CD27+/-, CD95+)	18,2 (15,15 - 46,88)	31,5083 (0 - 49,37)	0,917
% CD8 Peripheral memory (CD3+, CAR+, CD8+, CD4-, CD45RA-, CD62L-, CD27+/-, CD95+)	42,4242 (13,33 - 58,33)	39,9853 (0 - 67,34)	0,754
% CD8 Inmadure effector (CD3+, CAR+, CD8+, CD4-, CD45RA+, CD62L+low, CD27+low, CD95+)	0 (0 - 1,52)	2,51 (0 - 10,68)	0,126
% CD8 Intermediate effector (CD3+, CAR+, CD8+, CD4-, CD45RA+, CD62L-, CD27+low, CD95+)	0 (0 - 19,7)	5,74 (0 - 9,55)	0,666
% CD8 Final effector (CD3+, CAR+, CD8+, CD4-, CD45RA++, CD62L-, CD27-, CD95+)	0 (0 - 10,61)	0,81 (0 - 7,54)	0,22
**CAR-T CD4+** (CD3+, CD4+, CD8-) **cell/µL**	2,955 (0,88 - 7,11)	10,1395 (0,29 - 240,08)	0,465
% CD4 Naive (CD3+, CAR+, CD4+, CD8-, CD45RA+, CD62L+, CD27++, CD95-)	0 (0 - 0)	0,0675 (0 - 7,48)	0,116
% CD4 Central memory (CD3+, CAR+, CD4+, CD8-, CD45RA-, CD62L+, CD27+/-, CD95+)	36,6667 (12,5 - 50)	10,1012 (0 - 46,51)	0,285
% CD4 Peripheral memory (CD3+, CAR+, CD4+, CD8-, CD45RA-, CD62L-, CD27+/-, CD95+)	58,3333 (0 - 87,5)	18,0907 (0 - 76,36)	0,281
% CD4 Effector (CD3+, CAR+, CD4+, CD8-, CD45RA+, CD62L+, CD27-, CD95+)	0 (0 - 40)	0 (0 - 17,44)	1
**CAR-T CD8+ CD4+** (CD3+, CAR+, CD8+, CD4+) **cell/µL**	1,182 (0,57 - 1,82)	0,253 (0 - 43,52)	0,197
**CAR-T CD8- CD4-** (CD3+, CAR+, CD8-, CD4-) **cell/µL**	2,051 (0,46 - 6,22)	0,2915 (0 - 6,46)	0,272

Supplementary table 4: NK cells, B lymphocytes and other subpopulations of immune system analysed on day +28 post CAR-T and phenotype.

Cell type at day +28 after CAR-T	Non-relapses after CAR-T N=13<br>Median (range)	Relapses after CAR-T N=7<br>Median (range)	P
B cell (CD3-, CD56-, CD19+) cell/µL	*0 (0 - 0)*	*0 (0 - 0)*	*1*
**NK cell** (CD3-, CD56+) **cell/µL**	159,6735 (34,41 - 336,07)	64,372 (4,94 - 268,94)	**0,03**
% NK CD56 low (CD3-, CD56+low)	13,2877 (3,07 - 31,14)	18,3672 (4,05 - 72,13)	0,598
% NK CD56 bright (CD3-, CD56++)	88,8054 (52,09 - 96,93)	81,6328 (27,87 - 95,95)	0,468
**Eosinophils** (CD14-, CD16- SSChi) **cell/µL**	153,2325 (18,96 - 6799,07)	82,153 (19,39 - 546,04)	0,356
**Neutrophils** (CD14-, CD16 -/+) **cell/µL**	897,508 (97,46 - 2536,63)	709,614 (124,34 - 1216,59)	0,21
**Neutrophils CD16-** (CD14+, CD16-) **cell/µL**	3,174 (0 - 15,47)	2,45 (0 - 10,5)	0,947
**Basophils** (HLADR-, CD123++) **cell/µL**	45,642 (0 - 101,38)	20,7265 (1,55 - 104,04)	0,391
**Monocytes** (CD4+, CD11c+, HLADR+) **cell/µL**	410,778 (127,01 - 648,19)	326,124 (35,32 - 603,32)	0,429
% Classic monocytes (CD14+ CD16-)	69,7611 (47,31 - 87,34)	68,0815 (46,74 - 78,21)	0,468
% Intermediate monocytes (CD14+ CD16+)	13,0234 (4,09 - 24,63)	14,5113 (10,15 - 26,82)	0,21
% No classic monocytes (CD14- CD16+)	15,702 (6,73 - 31,15)	18,8073 (6,62 - 28,86)	0,434
**plasmacytoid dendritic cells** (CD4+, HLADR+, CD11c-, CD123++) **cell/µL**	4,1025 (0 - 12,88)	5,815 (0,15 - 15,25)	0,644
**Mieloid dendritic cells** (CD4+ HLADR+ CD11c+, CD123low) **cell/µL**	9,131 (2,21 - 25,02)	10,0485 (0,59 - 46,92)	0,895

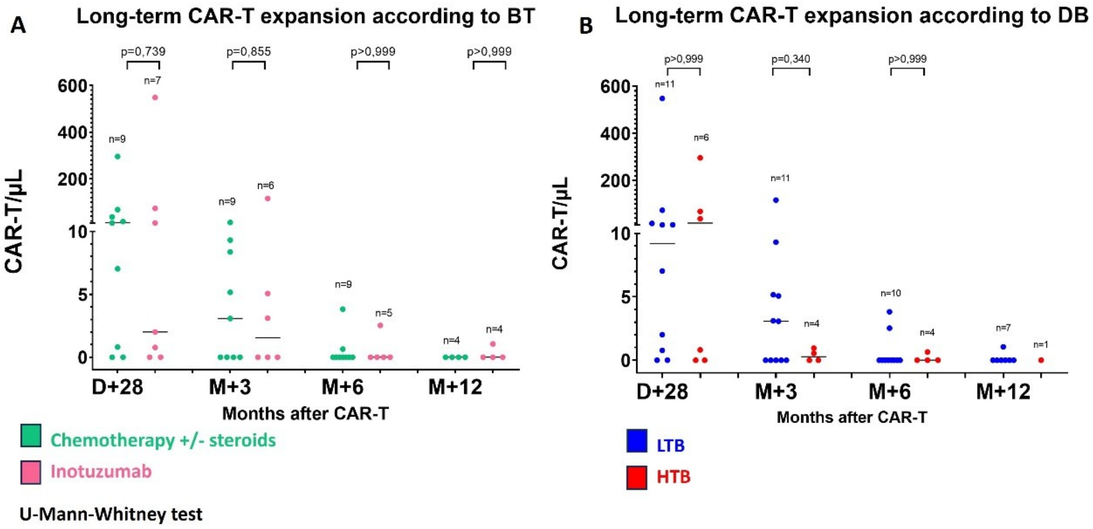

Supplementary figure 5: CAR-T cell persistence according to BT and disease burden*: No differences in expansion were observed between the groups based on Bridge Therapy (BT) or tumor burden. A p-value of 0.05 was considered statistically significant, and the non-parametric Mann-Whitney test was employed*

Supplementary table 5: CAR-T cell expansion correlated with tumor burden and residual B-cell population prior to CAR therapy and post-BT.

	LTB		HTB	
	CAR/µL peak of expansion	B-cells % + Tumor Burden %	CAR/µL peak of expansion	B-cells % + Tumor Burden %
**Inotuzumab group**				
	76.1	15.7% + 1.3%	59.2	0.004+ 34.1%
	23.2	0% + 0%	20889.1	0% + 0.9% + high EM
	49.3	0.002% + 0%		
	58.3	0.05% + 0%		
	681.67	4.3% + 0.2%		
	33.54	0% + 0%		
	71.69	0% + 0%		
	3.27	0% + 0%		
**chemotherapy +/- steroids**				
	825	4.9% + 2.5%	149.5	16.8% + 20.9%
	190.4	6.1% + 0.6%	638.9	2.2% + 40.9%
	145	0.1% + 0%	441	0% + 14.2%
	1537.1	1.1% + 0.035%	904.4	0.7% + 6.6%
	204.6	0.15% + 0.14%	2231.7	0.01% + 78.8%
	234.45	0.1% + 2.9%	2392.7	0% + 97.5%
	146	0.08% + 0		
	100.88	0.12% + 0.01%		

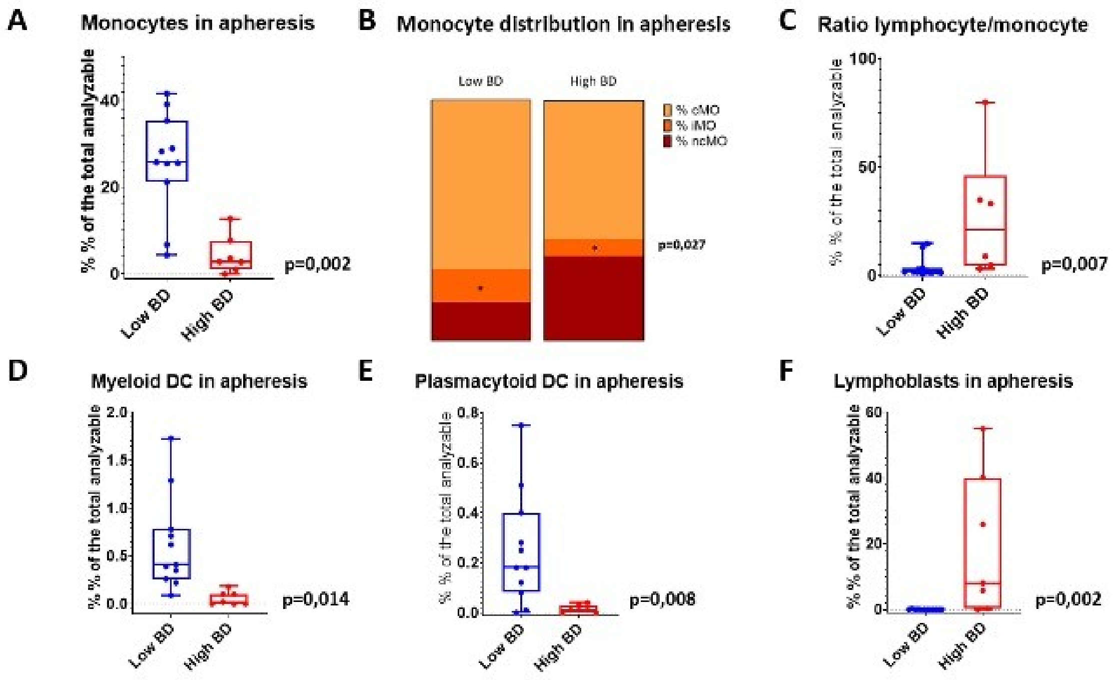

Supplementary figure 6: Characteristics of apheresis (A) percentage of monocytes from total cells of leukapheresis, (B) distribution of monocyte subpopulations (cMO: classical, iMO: intermediate, ncMO: non-classical), (C) ratio of lymphocyte/monocytes, (D) percentage of myeloid DC (DC: denditrics cells), (E) percentage of plasmocytoid dendritic cells and (F) percentage of lymphoblasts in apheresis. Statistical differences between groups were assessed using Mann-Whitney test. Differences are considered significant ($p<0.05$) and shown in bold.
